# More Light Sought

**Published:** 1875-02

**Authors:** 


MORE LIGHT SOUGHT.—One of our subscribers, who takes great interest in matters of diet, and indeed in all topics connected with human life and domestic economy, suggests that we solicit from our thoughtful readers the result of their experience and observation, pro and con, upon the following questions: Are two meals a day better than three? Is salt necessary for man or beast? Is meat a healthful and economical food? Is cheese valuable as an article of diet, and not merely as a luxury? The value of heat as a remedial agent.

We cheerfully respond to this suggestion, and will thank our friends who are able to bear testimony upon any of these points, and who take interest enough in them to feel willing to enlighten the world through our pages, to send us short communications on all these subjects, as well as upon all others which fall within the range of topics discussed by our magazine.

There are other subjects which we would be glad to open our pages to, if our many intelligent readers will lend us their aid. We believe we can do no greater good than by devoting some of our time and space to the treatment of the diseases of the horse—that speechless slave—who serves us so faithfully. Will not our friends, who sympathise with these bearers of our burdens, tell us how the ills which ordinarily afflict them may be speedily relieved? And will not experienced persons give us their views on the modern practice of clipping horses in winter, a system which compels them to face the chilling blasts practically unclad? To us it appears to be an abomination, opposed to nature and not far from brutal; and we should be glad to get the opinions of others on the subject.

---

You don't need undershirts half as much as you need drawers. The chest gets protection enough usually; it is the legs which are badly clothed.

